# Psychosocial job characteristics, wealth, and culture: differential effects on mental health in the UK and Thailand

**DOI:** 10.1186/s12992-015-0116-x

**Published:** 2015-07-08

**Authors:** Vasoontara Yiengprugsawan, Antonio Ivan Lazzarino, Andrew Steptoe, Sam-ang Seubsman, Adrian C Sleigh

**Affiliations:** National Centre for Epidemiology and Population Health, Research School of Population Health, ANU College of Medicine, Biology and Environment, The Australian National University, Building 62 Mills Road, Acton 2601 Canberra, Australia; Department of Epidemiology and Public Health, University College London, 1-19 Torrington Place, London WC1E 6BT, UK; School of Human Ecology, Sukhothai Thammathirat Open University, Nonthaburi, Thailand

**Keywords:** Psychosocial factors, Mental health, Job characteristics, UK, Thailand

## Abstract

**Background:**

Most research on the influence of psychosocial job characteristics on health status has been conducted within affluent Western economies. This research addresses the same topic in a middle-income Southeast Asian country, enabling comparison with a Western benchmark.

**Methods:**

We analysed and compared the Health Survey for England conducted in 2010 and the Thai Cohort Study data at 2005 baseline for workers aged 35–45 years. Multivariate logistic regression was used to assess relationships between psychosocial job characteristics and health, measured as Adjusted Odd Ratios (AOR), controlling for potential covariates in final analyses.

**Results:**

In both UK and Thai working adults, psychological distress was associated with job insecurity (AOR 2.58 and 2.32, respectively), inadequate coping with job demands (AOR 2.57 and 2.42), and low support by employers (AOR 1.93 and 1.84). Job autonomy was associated with psychological distress in the UK samples (AOR 2.61) but no relationship was found among Thais after adjusting for covariates (AOR 0.99). Low job security, inability to cope with job demands, and low employer support were associated with psychological distress both among Thai and UK workers.

**Conclusions:**

Job autonomy was an important part of a healthy work environment in Western cultures, but not in Thailand. This finding could reflect cultural differences with Thais less troubled by individualistic expression at work. Our study also highlights the implications for relevant workplace laws and regulations to minimise the adverse job effects. These public health strategies would promote mental health and wellbeing in the population.

## Introduction

In Western society there is already a considerable body of evidence associating the psychosocial environment at work with overall health and wellbeing [1]. In particular, low job control and associated stress have been reported as determinants of all-cause mortality based on prospective longitudinal studies [2]. These adverse psychosocial work factors have also been reported to affect morbidity, such as risk of coronary heart disease [3]. In addition, chronic work stress can increase the risk of fatigue and depression [4].

Common characteristics of adverse psychosocial workplace factors include job insecurity, job strain with high demands and job autonomy, long work hours, shift work, and overtime [1, 5]. These adverse workplace factors were also reported to associate with negative lifestyle health-risk behaviours such as physical inactivity, smoking, and alcohol consumption [6]. The protective and buffering role of social support at work on adverse health outcomes has been noted in diverse settings [7].

Most research on the influence of psychosocial job characteristics on health status has been conducted within affluent Western economies, although a few studies have been carried out in high-income areas of Asia. The Asian reports include a cross-sectional study linking psychosocial work factors and health among Taiwanese [8] and a prospective cohort study of Japanese workers that psychological demands and work engagement affect health [9]. In middle-income Southeast Asian countries, there has been some research on psychosocial work environments [10] but there is limited information on psychosocial work-health relationships.

The aim of our research reported here is to provide evidence about the relationship between psychosocial job characteristics (security-demands-autonomy-support) and psychological health in a middle-income Southeast Asian country, in comparison with a Western benchmark. We use data from a large national cohort study in Thailand and the Health Survey for England. Thailand is going through an economic transition as well as a transition from traditional illnesses (maternal and child mortality and infectious diseases) to chronic diseases, including psychological health problems. The Thai workforce is rapidly formalising as the country industrialises and our results will help to address the knowledge gap regarding psychosocial work conditions in transitional middle-income Asia. We also describe differences in work-health relationships between Thailand and the UK and explore the relative importance of wealth and culture.

## Methods

### Data source

This study was based on two datasets: the Thai Cohort Study and the Health Survey for England [11]. Details of data harmonisation are as follows:

- **The Health Survey for England (HSE)** is a nationally representative survey conducted periodically since 1991. It covers individuals aged 16 years and over in private households across England using stratified random sampling. The HSE has core modules that are used every year and special topics introduced in selected years. Core health questions include: general health, fruit and vegetable intakes, height and weight, tobacco and alcohol consumption, and certain illnesses. In selected years, other conditions include oral health, chronic diseases, and physical activity. This study is restricted to the 2010 HSE which contains comprehensive questions on psychosocial work conditions.
- **The Thai Cohort Study (TCS)** is part of the overarching Thai Health-Risk Transition: A National Cohort Study. The study consists of 87,134 adults enrolled at the Sukhothai Thammathirat Open University (STOU) who volunteered to participate in the 20-page health questionnaire covering socio-geo-demographic characteristics, self-reported physical and mental health and personal wellbeing, weight and height, health-risk behaviours including tobacco and alcohol consumption, diet and exercise, as well as health service use. The TCS involved two subsequent follow-ups in 2009 and 2013, with response rates of over 70 %. The TCS represents well the sex ratio, geographic location, religion, and modest income of working Thai adults [12]. Our report presented here is restricted to the 2005 baseline TCS questionnaire which provides a wide array of questions on psychosocial job characteristics.

### Data harmonisation

This section briefly summarises our approach to preparing the TCS and HSE data for comparative analyses. Full details of the harmonised variables are included in Appendix 1. In order to enable comparison of the relationship between psychosocial job characteristics and health between the two datasets, we proceeded as follows:

1. **Restriction of age and paid work distribution**: we found that between the two datasets, respondents aged between 35 and 45 years had the highest (and most similar) proportion of paid workers (Table 1 – paid work for 35–45 year-olds reported by 70 % of 1618 in the UK and 88 % of 19843 in TCS). Our analyses were therefore restricted to working adults in that age group resulting in 1,132 HSE and 17,459 TCS respondents. By doing this, we avoided age confounding and maximised statistical power by using the age band with the most paid workers. This age group is particularly suitable for analysis because it includes individuals well established in their workplaces, and furthermore the participants were approaching the years at which chronic diseases emerge.
2. **Psychological distress:** In the HSE, psychological distress was assessed using the 12-item General Health Questionnaire [13]. In the TCS, we used the Kessler psychological distress inventory [14] which included three items assessing the level of anxiety over the past four weeks. In both datasets, we summed and recoded responses to create a dichotomous variable for psychological distress (Appendix). Both GHQ and Kessler measure general psychological distress but neither measure has questions about job-related stress.
3. **Job factors:** there were four psychosocial job characteristics in this study: security, autonomy (decision at work), ability to cope with demands, and support by employer. Details of questions and distribution of responses are reported in Appendix 1 and Table 2.
4. **Other covariates:** in order to examine the relationship between job characteristics and psychological distress, we have taken into account socioeconomic status proxied by occupations. In both TCS and UK HSE, we divided occupational status into three groups: managerial and professional, intermediate/office workers, and skilled or manual workers. In addition, we included information on part- or full-time paid work. Analyses were also adjusted for health-risk behaviours (smoking and drinking) which were dichotomised (yes or no) in both datasets.

**Table 1:** Distribution of age, work status, and key variables among UK and Thai source populations

Key variables	Health Survey for England (*n* = 14112)		Thai Cohort Study (*n* = 87134)	
Age groups	Age distribution^a^	In paid work	Age distribution	In paid work
<20	43.2 (6093)	3.1 (122)	2.9 (2502)	1.6 (1151)
20–34	11.6 (1642)	25.5 (1014)	68.7 (59894)	68.4 (48690)
35–45	11.5 (1618)	28.5 (1132)^b^	22.8 (19843)	24.5 (17459)
46–65	20.1 (2837)	40.4 (1606)	5.5 (4792)	5.4 (3820)
65+	13.6 (1922)	2.5 (98)	0.1 (103)	0.04 (31)
**Psychological distress**				
High distress	11.7 (132)		12.3 (3857)	
**Job security**				
Extremely secure	28.0 (317)		18.5 (5726)	
Secure	40.6 (459)		50.1 (15553)	
Moderately secure	19.5 (221)		21.5 (6667)	
Not at all secure	11.9 (135)		10.0 (3094)	
**Autonomy**				
Often	48.5 (547)		55.1 (16996)	
Sometimes	35.3 (398)		33.4 (10295)	
Rarely	11.9 (135)		9.1 (2807)	
Never	4.2 (47)		2.5 (760)	
**Coping with demands**				
Often	38.9(438)		48.2(14834)	
Sometimes	49.1(553)		40.5 (12473)	
Rarely/never	12.0(135)		11.3 (3475)	
**Support by employer** ^**a**^				
Often	39.9(449)		58.4(17937)	
Sometimes	34.6 (389)		23.8 (7309)	
Rarely/never	12.5 (140)		8.7 (2680)	
Not applicable	12.9 (145)		9.1 (2790)	
**Occupation groups**				
High	53.1 (597)		25.3 (6703)	
Medium	18.8 (18.8)		44.3 (11758)	
Low	28.2 (28.2)		30.4 (8059)	
**Hours of paid work**				
35 hours + per week	73.5 (832)		84.5 (26397)	

^a^Column % (n) for all columns

^b^For this age group, paid work reported by 70 % (1132/1618) in UK and 88 % (17459/19843) in Thailand

**Table 2:** Associations between job variables and psychological distress comparing the UK and Thailand

Job factors and covariates	Odds Ratios for psychological distress^a^ [95 % Confidence Interval]			
	Heath Survey for England (*n* = 1132)		Thai Cohort Study (*n* = 17459)	
	Bivariate OR	Multivariate AOR^b^	Bivariate OR	Multivariate AOR^b^
**Job security**				
Extremely secure	1.00	1.00	1.00	1.00
Secure	1.46 [0.88–2.43]	1.44 [0.78–2.68]	1.08 [0.98–1.20]	1.02 [0.91–1.14]
Moderately secure	**2.30 [**1.32–3.98]	1.82 [0.94–3.53]	**1.71** [1.53–1.92]	**1.56** [1.37–1.77]
Not at all secure	**2.64** [1.44–4.84]	**2.58** [1.28–5.22]	**2.82** [2.49–3.18]	**2.32** [2.00–2.69]
**Autonomy**				
Often	1.00	1.00	1.00	1.00
Sometimes	**2.31** [1.51–3.54]	**1.78** [1.07–2.98]	0.89 [0.82–0.96]	0.79 [0.72–0.86]
Rarely	**2.67** [1.54–4.66]	**2.16** [1.14–4.10]	**1.37** [1.23–1.53]	0.97 [0.85–1.10]
Never	**3.52** [1.63–4.61]	**2.61** [1.09–6.25]	**1.57** [1.30–1.90]	0.99 [0.79–1.24]
**Coping with demands**				
Often	1.00	1.00	1.00	1.00
Sometimes	**1.69** [1.08–2.65]	1.33 [0.79–2.25]	**1.37** [1.27–1.48]	**1.34** [1.22–1.46]
Rarely/never	**5.14** [3.05–8.68]	**2.57** [1.35–4.92]	**2.64** [2.39–2.90**]**	**2.42 [**2.17–2.71]
**Support by employer**				
Often	1.00	1.00	1.00	1.00
Sometimes	**1.81** [1.16–2.83]	1.44 [0.89–2.33]	**1.52** [1.41–1.65]	**1.36** [1.24–1.49]
Rarely/never	**3.06** [1.68–5.57]	**1.93** [1.05–3.56]	**2.35** [2.12–2.62]	**1.84** [1.63–2.08]
**Covariates**				
**Sex**				
Males		1.00		1.00
Females		1.21[0.75–1.95]		1.09 [0.99–1.19]
**Occupational status**				
High		1.00		1.00
Medium		0.72 [0.44–1.19]		0.95 [0.86–1.05]
Low		0.73 [0.44–1.21]		1.02 [0.94–1.05]
**Part-time/full-time**				
Part-time		1.00		1.00
Full-time		1.36 [0.83–2.23]		1.16 [0.95–1.29]

^a^In the Health Survey of England, we assessed psychological distress using the 12-item General Health Questionnaire; in the Thai Cohort Study, we used the Kessler psychological distress inventory. Full details of the harmonised variables are included in Appendix 1; this includes three items assessing the level of anxiety over the past four weeks. Results in **bold font** were statistically significant at *p* < 0.05

^b^All job factors and covariates are mutually adjusted in the multivariable model

### Data analysis

We first describe the distribution of age and paid work status by country of residence. Subsequent analyses were restricted to the age group that had respondents with similar proportion of paid work (35–45 years). Key variables such as psychological distress, psychosocial job characteristics, and other covariates are detailed in each dataset. Bivariate logistic regression was used to calculate Odd Ratios (OR) and 95 % Confidence Intervals (CI) for the association between psychological distress and job conditions in each dataset. Multivariate logistic regression was used to provide Adjusted Odd Ratios (AOR) and 95 % CI, controlling for covariates in the final analyses.

### Ethics

The TCS ethics approval was obtained from Sukhothai Thammathirat Open University Research and Development Institute (protocol 0522/10) and the Australian National University Human Research Ethics Committee (protocols 2004/344 and 2009/570). The UK HSE ethics approval was obtained from the London Research Ethics Committee.

## Results

There was a total of 14,112 participants in the Health Survey for England in 2010 and 87,134 respondents for the baseline Thai Cohort Study in 2005. The distribution of participants by work categories and by age groups is described in Table 1; paid work respondents were proportionally highest for the age group 35 to 45 years and comparative analyses were restricted to this age group (see Methods).

The exposures of interest were the psychosocial job characteristics detailed in Table 2. These factors were distributed as follows: low job security affected about one tenth of the UK and Thai samples (11.7 % vs 12.3 %); low job autonomy was noted by 4.2 % of UK and 2.5 % of Thai respondents; ‘rarely or never’ coping with job demands affected about 10 % of respondents in both populations; and low support from employers was reported by 12.5 % among the UK and 8.7 % of the Thai respondents. The health outcome (psychological distress) afflicted about 12 % of respondents in each of the two settings (Table 1 and Appendix).

Associations between psychosocial job characteristics and psychological distress are reported in Table 2, stratified by the two study groups. Bivariate and multivariate results are shown so as to enable assessment of confounding. The multivariate odds ratios were as follows. Low job security strongly associated with psychological distress in both settings – AOR 2.58 [95 % CI 1.28–5.22] in the UK and AOR 2.32 [95 % CI 2.00–2.69] in the Thai respondents. Inability to cope with work demands was also strongly associated with psychological distress in both groups (AOR 2.57 and 2.42 among the UK and Thai samples), as was low support from employers (AOR 1.93 and 1.84 among the UK and Thai workers). However, there was a clear difference regarding job autonomy in the two settings: UK respondents with low decision capacity at work were highly likely to report psychological distress (AOR 2.61 95 % CI 1.09–6.25), but no such association was found in the Thai group after covariates had been taken into account.

To investigate this issue of discordant UK-Thai job autonomy effect on psychological health, we repeated the Thai analyses restricting to the upper 20 % or upper 10 % according to income or (separately) wealth (based on replacement values for a standard basket of household assets). The income-restricted estimates showed a modest (but not statistically significant job autonomy effect) ranging from AOR 1.17 for ‘rarely’ to AOR 1.44 for ‘never’ which was attenuated as income fell and disappeared altogether when restrictions were lifted. But there was no effect when analyses were restricted by wealth. We cautiously conclude that it is unlikely that economic differences between the UK and Thailand explain the lack of a job autonomy effect on psychological health of Thais. It is more plausible that that this difference could be related to other factors such as culture and religion.

## Discussion

Our international comparison provides evidence of substantial and statistically significant associations between certain psychosocial job characteristics and psychological distress among both UK and Thai middle-aged working adults. Low job security, inadequate coping with job demands, and low support from employers were associated with psychological distress among working adults in both countries. However, it is also notable that a fourth psychosocial job factor – low autonomy – was strongly associated with adverse psychological health outcomes in the UK workers but that such as association was not apparent among Thais.

Our results support previous studies on association between psychosocial job characteristics and health [8, 9]. The importance of employer support reported in our study is consistent with other studies in Asia, in particular, as noted by a study in the neighbouring middle-income country of Malaysia [10]; in that setting emerging global forces in the economy will increase job demands and could reduce support at work, thus further highlighting the importance of the protective role of employer support at work [15, 16].

The noteworthy difference between the UK and Thailand was the effect of low job autonomy. Job autonomy or control is thought to be a particularly important aspect of a healthy work environment in Western cultures, and is associated with greater job satisfaction and favourable biological responses [17]. This effect could plausibly be related to levels of economic development or to the influence of East-west differences in cultural factors. About a quarter of the Thai respondents were working at a professional or managerial level, whereas over half of the UK respondents were in that category. The difference in occupational status could account for the lack of importance of low job autonomy for Thai respondents. However, it should also be noted that Buddhist religious sentiment was reported to be important for almost all cohort members and this attitude and tolerance could boost acceptance of low job autonomy.

### Strengths and limitations of this study

The strength of our study is the comparative analyses of psychosocial job characteristics and associated psychological distress between the two very different countries. Data harmonisation was possible producing similar categories for job conditions, psychological distress, and other covariates. In addition, the design of the questionnaires avoided any suggestions about relationships between work factors and health in both the Thai and UK studies; queries about work and heath appeared in different sections of the questionnaire without any hint of our underlying hypotheses.

However, we also note some limitations of this study. Our analysis was cross-sectional in nature and does not establish causal relationships between job factors and health. In addition, the Health Survey for England participants were nationally representative but the Thai participants were all adult students enrolled in an Open University and residing nationwide. In order to produce more homogenous groups for analyses, we restricted our study to middle-aged working adults and thus our results are not representative to general population. It should be noted that the 12-item General Health Questionnaire measures (UK) both anxiety and depression, the three items from the Kessler Psychological Distress measures focuses on anxiety (Thailand), however our harmonised cut-offs have captured the high distress group in both datasets (approximately 12 %). We also performed sensitivity analyses excluding UK participants who had not completed high school but the results were similar (data not shown).

### Future research

Our cross-country comparisons capture work-health relationships in two very different settings, Thailand and the UK. For the fourth job feature – autonomy – we found a UK-Thai difference with Thais apparently unaffected. This finding could reflect cultural differences with Thais less troubled by individualistic expression at work. We have speculated that differences in culture and religion may explain the lack of a job autonomy effect on psychological health among Thais. Cross-national qualitative ethnographic investigation could expand knowledge on the role of culture operating on occupational mental health.

### Implications of the study

Empirical evidence on East-west cross-national comparisons such as ours is very limited, and this is particularly true when comparing high and middle income countries. Such comparisons are, however, very important for understanding the cultural context of public health policies, and the relevance of Western concepts to other cultures. The psychological health of Thai and UK respondents was equally affected by three psychosocial job features – security, coping with demands, and employer support. Our study also highlights the implications for relevant workplace laws and regulation, including providing workers with constructive performance feedback as well as regular consultation and training for managing workplace stress to minimise the adverse job effects. These public health strategies would promote mental health and wellbeing in the population. These issues will be of increasing importance in Thailand and similar transitional countries in Asia as the workforce formalises. However, each country needs to examine the issues operating within its own cultural context and cannot simply import information from other settings.

## Appendix 1

**Table 3:** Harmonising the data from the UK Health Survey for England and the Thai Cohort Study

	UK Health Survey for England		Thai Cohort Study (TCS)	
	Original format	UK-TCS harmonised	Original format	TCS-UK harmonised
Psychological distress	General Health Questionnaire-(GHQ) 12 questions:^a^ able to concentrate, lost much sleep over worry, felt playing useful part in things, felt capable of making decisions, felt constantly under strain, felt could not overcome difficulties, able to enjoy normal day-to-day activities, been able to face problems, been feeling unhappy and depressed, been losing confidence in self, been thinking of self as worthless, been feeling reasonably happy	Final binary outcome: ^a^	Kessler Psychological distress: (three questions)	Final binary outcome:
		High distress score 4+: 11.7% (132)	In the past 4 weeks, about how often… 1) did you feel nervous? 2) did you feel restless and fidgety? 3) did you feel that everything was an effort?	High distress (all/most of the time) 12.3% (3,857)
			Responses on a five-point scale: ‘all of the time’, ‘most of the time’, ‘some of the time’, ‘little of the time’, ‘none of the time’.	
			‘All’ or ‘most’ of the time coded as having high distress; rest code as no serious problem.	
Job security	How likely is it that you will lose your job and become unemployed within the next twelve months? Please estimate the probability of such a chance on a scale from 0 to 100. (0 means that such a change will definitely not take place. 100 means that such a change definitely will take place.)	Collapsed response categories:	How secure do you feel about your job or career future on your current occupation?	Response categories: Extremely secure 18.5% (5726)
		0 28.0% (317) ^b^		Secure 50.1% (15553)
		10–30 40.6% (459)	- Extremely secure	Moderately secure 21.5% (6,667)
		40–60 19.5% (221)	- Secure	Not at all secure 10.0% (3,094)
		>60 11.9% (135)	- Moderately secure	
			- Not at all secure	
Decision at work	Do you have a choice deciding how you go about your work?	Collapsed response categories:	I have a good deal of say in decisions about work	Response categories:
				Often 55.1% (16,996)
		All/most of the	- Often	Sometimes 33.4% (10,295)
	- All of the time	time 48.5% (547)	- Sometimes	
			- Rarely	Rarely 9.1% (2,807)
	- Most of the time	Much/some of the time 35.3% (398)	- Never	Never 2.5% (760)
		Occasionally 11.9% (135)		
		Never 4.2% (47)		
	- Much of the time			
	- Some of the time			
	- Occasionally			
	- Never			
Coping with demands	How much do you agree or disagree with the statement that ‘I feel able to cope with the demands of my job’?	Collapsed response categories:	Your work situation… I have enough time to do everything	Response categories:
		Often (strongly agree) 38.9% (438)	- Often	Often 48.2% (14,834)
			- Sometimes	Sometime 40.5% (12,473)
			- Rarely	Rarely/never 11.3% (3,475)
	- Strongly agree	Sometime (agree) 49.1% (553)	- Never	
	- Agree			
	- Neither agree nor disagree	Rarely/never 12.0% (135)		
		(neither agree nor disagree/		
	- Disagree	disagree/strongly disagree)		
	- Strongly disagree			
Support from employers	Do you get help and support from your line manager?	Collapsed response categories:	How would you rate the support you are getting from employer/boss?	Collapsed response categories:
	- Often	Often 39.9% (449)	- A lot of support	Often (lot/quite a bit) 58.4% (17,937)
	- Sometimes		- Quite a bit of support	
	- Seldom	Sometimes 34.6% (389)	- A little support	Sometimes (little) 23.8% (7,309)
	- Never	Seldom/never 12.5% (140)	- Very little support	Seldom (very little) 8.7% (2,680)
	- Does not apply/have no manager	Does not apply 12.9% (145)	- Not relevant	Not apply (not relevant 9.1% (2,790)
Occupation status	National Statistics Socio-economic Classification	Response categories:	Current occupation	Response categories:
	- managerial and professional	High 53.1% (597)	professionals/managers	High 25.3% (6,703)
	- intermediate	Medium 18.8% (211)	- office assistants	Medium 44.3% (11,758)
	- routine and manual	Low 28.2% (317)	- skilled and manual workers	Low 30.4% (8,059)

^a^GHQ-12: UK General Health Questionnaire with 12 items; each item categorised on a four-point scale later collapsed to a positive/negative binary result. The negative binary response (0) includes ‘not at all’ or ‘same as usual’ and the positive binary response (1) includes ‘more than usual’ or ‘much more than usual’. The sum of binary responses across the 12 items was then collapsed, the binary total for each individual was divided into <4 (no serious problems) and 4+ (serious problems) for final comparative analyses

^b^This is the smallest proportion of the UK total that could reasonably to be classified as ‘extremely secure’
